# Lung water, aeration and function in late preterm/term neonates: subgroup analysis of the ULTRAS study

**DOI:** 10.1038/s41390-025-04226-3

**Published:** 2025-06-26

**Authors:** Francesco Vinci, Barbara Loi, Luca Ramenghi, Daniele De Luca

**Affiliations:** 1 Department of Neuroscience, Ophthalmology, Genetics and Mother-Child Health, University of Genoa, Genoa, Italy; 2 Division of Paediatrics and Neonatal Critical Care, “A. Béclère” Hospital, APHP-Paris Saclay University, Paris, France; 3 Physiopathology and Therapeutic Innovation Unit-INSERM U999, Paris Saclay University, Paris, France

## Abstract

**Background:**

We aimed to study the pathophysiology of late preterm and term neonates with respiratory failure, without lung inflammation, and identify features associated with surfactant treatment.

**Methods:**

Ancillary subgroup analysis of a prospective multicenter cohort study. Forty-eight neonates were enrolled. Extravascular lung water (estimated as thoracic fluid content (TFC)), lung ultrasound score (LUS) and extended LUS (eLUS), transcutaneous partial pressure of carbon dioxide (PtcCO_2_) and O_2_ (PtcO_2_), fraction of inspired oxygen (FiO_2_), preductal peripheral hemoglobin saturation (SpO_2_) and derived metrics (SpO_2_/FiO_2_, oxygenation index (OI) and oxygen saturation index (OSI)) were assessed.

**Results:**

TFC was similar between neonates treated (13.2 [10.7–16.6] KOhm^−1^/Kg) and not treated with surfactant (15.6 [12.9–19.7] KOhm^−1^/Kg, *p* = 0.197). Lung aeration was better in neonates who did not need surfactant (LUS = 6 [4–8]; eLUS = 11 [9–16]) than in those who did (LUS = 10 [10–12], *p* < 0.001; eLUS = 19 [17–20], *p* < 0.001). PtcCO_2_ was similar between groups. Oxygenation was better in neonates who did not receive surfactant (PtcO_2_/FiO_2_ ratio = 281 [211–346]; SpO_2_/FiO_2_ ratio = 445 [388–462]; OI = 2.1 [1.6–2.6]; OSI = 1.3 [1.3–1.5]) than in those who did (PtcO_2_/FiO_2_ = 168 [110–244], *p* = 0.003; SpO_2_/FiO_2_ = 233 [183–288], *p* < 0.001; OI = 3.9 [2.5–5.4], *p* = 0.002; OSI = 2.6 [2.2–3.3], *p* < 0.001).

**Conclusions:**

Late preterm and term neonates with respiratory failure treated with surfactant show decreased lung aeration associated with impaired oxygenation likely due to surfactant insufficiency rather than excessive extra-vascular lung water.

**Impact:**

- Late preterm and term neonates receiving surfactant show decreased lung aeration and impaired oxygenation, likely due to surfactant insufficiency, rather than to excessive extra-vascular lung water.
- This helps characterising respiratory failure in late preterm and term neonates and identifying those who are at risk of surfactant insufficiency and need closer monitoring.

## Introduction

Surfactant replacement is the causal therapy for hyaline membrane disease (i.e. respiratory distress syndrome (RDS)) due to primary surfactant deficiency.^1,2^ The prevalence of RDS is inversely proportional to gestational age,^3^ thus, in late preterm or term neonates, respiratory failure may often be caused by mechanisms other than primary surfactant deficiency. For instance, the pathophysiology of transient tachypnoea of the neonate (TTN) is related to excessive alveolar fluid that fails to clear immediately after birth,^4^ although some patients with TTN may have co-existing relative surfactant deficiency.^5^

It is impossible to distinguish the pathophysiology based on clinical appearance only. Elucidating the respiratory pathophysiology may assist the clinicians in individualizing the clinical management and henceforth might prevent delayed administration of surfactant and complications, such as airleaks or pulmonary hypertension.^6,7^ Furthermore, many late preterm and term neonates are delivered in I/II level perinatal centers.^8^ A substantial cohort of these mature neonates may require advanced interventions for which they would have to be shifted to tertiary care neonatal intensive care units (NICU). Timely transfer of these neonates may reduce the burden on the healthcare system.^9^ Thus, the understanding of pathophysiology may also be important to reduce the downsides related to their transfer. Some recent minimally invasive technologies, such as electrical cardiometry and quantitative lung ultrasound, may help clarifying the pathophysiology of neonates presenting with similar clinical appearance. We aimed to apply them to late preterm and term neonates with respiratory failure to identify the features associated with surfactant treatment.

## Methods

### Study design

This was an ancillary project of an observational, international, multicenter study whose protocol is available in a public registry.^10^ The main results were recently published.^11^ This ancillary work was designed as a prospective cohort study based on subgroup analysis of the original population. All neonates were recruited, between December 2022 and November 2023, in only one center equipped with all the needed monitoring technologies (i.e. electrical cardiometry, quantitative lung ultrasound, and non-invasive complete gas exchange monitoring). The study was pragmatic as it used only data routinely obtained during clinical care which was not changed for study purposes. Ethical approval was granted (n.33/21) and parental consent was obtained as previously reported.^11^ The manuscript was prepared according to relevant guidelines.^12^

### Participants

Eligible patients were late preterm and term neonates (≥34^+0^ weeks’ gestation) with respiratory failure occurring within the first 72 h of life. For this subgroup analysis, all cases of respiratory failure due to lung infection or inflammation were excluded and we only considered neonates with a diagnosis of RDS or TTN, based on Montreux consensus integrated clinical criteria.^11^ Therefore, we excluded all neonates enrolled in the original study with a diagnosis of neonatal acute respiratory distress syndrome triggered by chorioamnionitis, perinatal asphyxia, infections and meconium or blood aspiration, as previously defined.^13,14^ The other exclusion criteria were the same of the original study,^11^ that is: major congenital malformations or chromosomal abnormalities, air leaks, need for surgery, hemodynamic instability (defined as need for any inotrope), congenital surfactant anomalies, pulmonary hypoplasia, pulmonary hypertension (defined as need for nitric oxide or other pulmonary vasodilators), and need for extracorporeal life support.

Respiratory support consisted of nasal mask–delivered, variable flow, continuous positive airway pressure (CPAP) set at 6 cmH_2_O. Supplemental oxygen was added if CPAP was insufficient to achieve pulsatile preductal peripheral hemoglobin oxygen saturation (SpO_2_) ≥90%. Poractant-alpha (200 mg/Kg) was administered, if the fraction of inspired oxygen (FiO_2_) was greater than 0.30,^15^ using a customized enhanced intubation-surfactant-extubation procedure.^16^ Other details of respiratory support policy were previously described^11,17^ and the remaining management was essentially based on current international guidelines.^18,19^

### Extra-vascular lung water estimation

Extra-vascular lung water was estimated using electrical cardiometry (Osypka Medical, San Diego, CA), whose principles of operation are described elsewhere.^20^ The technique has been previously used in neonates,^21,22^ and validated against the gold standard in adults under extra-corporeal circulation.^23^ Basically, it uses a high frequency, low amperage current applied through the thorax with 4 common electrocardiographic electrodes. The signal is affected by the thoracic impedance and interpreted considering the simultaneously captured electrocardiogram. Extra-vascular lung water was estimated as thoracic fluid content (TFC) and indexed to birth weight. TFC is calculated as the resistance to the passage of the electrical current due to total thoracic fluids and is correlated to the degree of pulmonary edema (i.e. the higher TFC, the greater the edema).^24,25^ The technique was used following the manufacturer’s recommendations^26^: electrodes for basic monitoring were placed as far as possible from those used by electrical cardiometry and measurements were considered only when a good signal was captured (quality index >80, in neonates lying still and supine, without electrocardiogram artifacts).^26^

### Lung aeration

Lung aeration was assessed with quantitative lung ultrasound using the lung ultrasound score (LUS) and the extended LUS (eLUS). Both scoring systems are based on classical semiology^27^ and are specifically validated for functional applications in newborn populations.^28^ LUS and eLUS were calculated on 6 and 10 (3 and 5 per each hemithorax) chest zones, and go from 0 to 18 or from 0 to 30 (i.e. the higher the value, the worse the aeration) for LUS and eLUS, respectively.^29,30^ eLUS includes the scan of posterior (dependent) lung zones by slightly tilting the neonate and slipping the probe between the thorax and the mattress.^30^ Micro-linear, “hockey-stick”, high (15 MHz) frequency probes (Philips, Eindhoven, The Netherlands) were used with machine settings as previously described.^31^ All scans were performed by neonatologists proficient in lung ultrasound on neonates quietly lying supine.

### Gas exchange

Gas exchange was assessed considering transcutaneously measured blood gases (PtcO_2_ and PtcCO_2_) and SpO_2_. Transcutaneous blood gases were measured with devices (Radiometer, Copenhagen, Denmark) appropriately calibrated at 44 °C,^13^ used according to American Association of Respiratory Care guidelines^32^ and manufacturer’s recommendations. These devices were calibrated using arterialized capillary blood gas analysis within our routine care. Pre-ductal SpO_2_ was measured on the right hand using an artifact filtering software (Nihon Kohden, Shinjuku, Japan) when the signal was regular and smooth. Oxygenation was described using PtcO_2_/FiO_2_ and SpO_2_/FiO_2_ ratios as well as modified oxygenation index (OI = mean airway pressure (Paw) × FiO_2_/PtcO_2_) and oxygen saturation index (OSI = Paw × FiO_2_/SpO_2_) where Paw was the CPAP level.

### Data collection and outcomes

TFC, PtcO_2_, PtcCO_2_ and SpO_2_ were averaged with 5” intervals over 1’ and recorded together with Paw, FiO_2_ and lung ultrasound scores. As per our clinical routine, electrical cardiometry, lung ultrasound and gas exchange evaluations were performed upon NICU admission, within 30’ from each other, and anyway before surfactant administration, if any. All data were recorded in real time. During all measurements, respiratory support was unchanged, and leaks were minimized by repositioning the infant and gently closing the mouth, as needed.^11^ Clinical and demographic data were extracted from electronic patient files.

TFC, lung ultrasound scores, PtcCO_2_, PtcO_2_/FiO_2_, SpO_2_/FiO_2_, OI and OSI were considered as study outcomes as they fairly analyze the whole pathophysiology cascade of neonatal respiratory failure.^33^ In detail, TFC assesses the extra-vascular lung water due to excessive alveolar fluid accumulation^4^ decreasing the air/fluid ratio. This ratio may however also be reduced by lung inflammation replenishing the alveoli of inflammatory fluid, albeit this was excluded because we only enrolled neonates with RDS or TTN. Lung ultrasound scores evaluate the air/fluid ratio by assessing the aeration, i.e. the lung volume available for gas exchange, without distinctions. Gas exchange metrics eventually describe the final consequences of the previous steps.

### Statistics

Data were compared with Student, Fisher or Mann–Whitney test, as appropriate. Correlation analyses were performed considering Spearman (ρ) coefficients and, if they were significant, adjusting them for gestational age using partial correlations. *p* < 0.05 was considered significant and analyses were performed with JASP (v.0.17.1; JASP Team (2023)).

## Results

Basic clinical details are described in Table 1. Neonates receiving surfactant were similar to those who did not receive it, with the exception of a higher proportion of male infants. NICU stay was obviously longer in surfactant treated-neonates (7 [6–10] days) than in those who did not receive the drug (4 [3–6] days, *p* = 0.04). Surfactant was administered at median postnatal age of 6 [3.5–18] hours of life. Nine and 39 neonates were diagnosed with RDS and TTN, respectively; all infants were successfully discharged, and no pulmonary hypertension or other complications were detected in any patient. The median time between data collection and surfactant administration, if any, was 42 min.

**Table 1:** Basic population characteristics.

	All neonates (*n* = 48)	Surfactant not given (*n* = 38)	Surfactant given (*n* = 10)	*p*
Gestational age (weeks)	35.6 (1.9)	35.5 (1.0)	36.2 (1.5)	0.269
Birth weight (g)	2696 (793)	2639 (843)	2915 (542)	0.332
Prenatal steroids	6 (12.5%)	6 (15.8%)	0	0.406
Caesarean section	26 (54.2%)	20 (52.6%)	6 (60%)	0.735
Male sex	33 (68.7%)	23 (60.5%)	10 (100%)	0.014
5’ Apgar score	9 [7–10]	9 [7–10]	9 [7–10]	0.736
SNAPPE-II score	5 [0–18]	5 [0–18]	6 [5–17]	0.311
Postnatal age at measurements (h)	6.2 [3–10]	6 [3–10]	6.5 [2.7–16]	0.698

Data are expressed as mean (standard deviation), number (%) or median [25^th^ – 75^th^ percentile] and compared with Student, Fisher or Mann–Whitney test, as appropriate. Prenatal steroids were defined as the administration of two 12-mg betamethasone doses, 24 h apart from each other, with the last given at least 24 h before the delivery. Postnatal age represents the moment when all measurements were performed. Apgar and SNAPPE-II are dimensionless variable.

*SNAPPE* score for neonatal acute physiology-II.

**Fig. 1:**
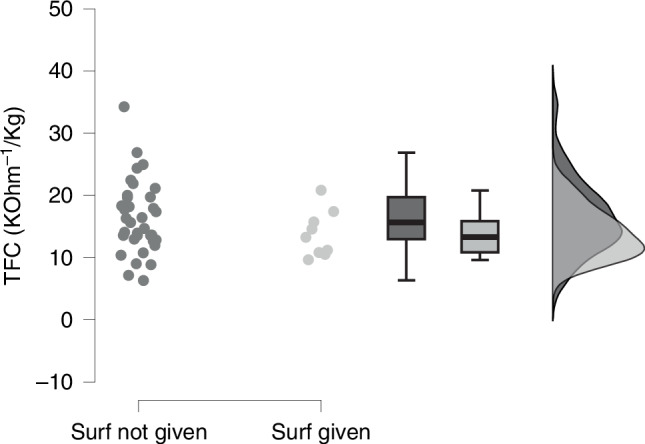
Extra-vascular lung water. Each dot represents a neonate; box plots depict (from top to bottom) 95^th^, 75^th^, 50^th^, 25^th^, and 5^th^ percentiles. The raincloud curves on the right side represent the density (distributions) of datapoints using two different gray tones. Data were analyzed with Mann–Whitney test. Surf surfactant, TFC thoracic fluid content.

The estimation of extra-vascular lung water is reported in Fig. 1. TFC was similar between neonates who received surfactant (13.2 [10.7–16.6] KOhm^−1^/Kg) and those who did not receive it (15.6 [12.9–19.7] KOhm^−1^/Kg, *p* = 0.197). TFC did not correlate with lung ultrasound scores or any gas exchange metrics, nor with the time of surfactant administration.

**Fig. 2:**
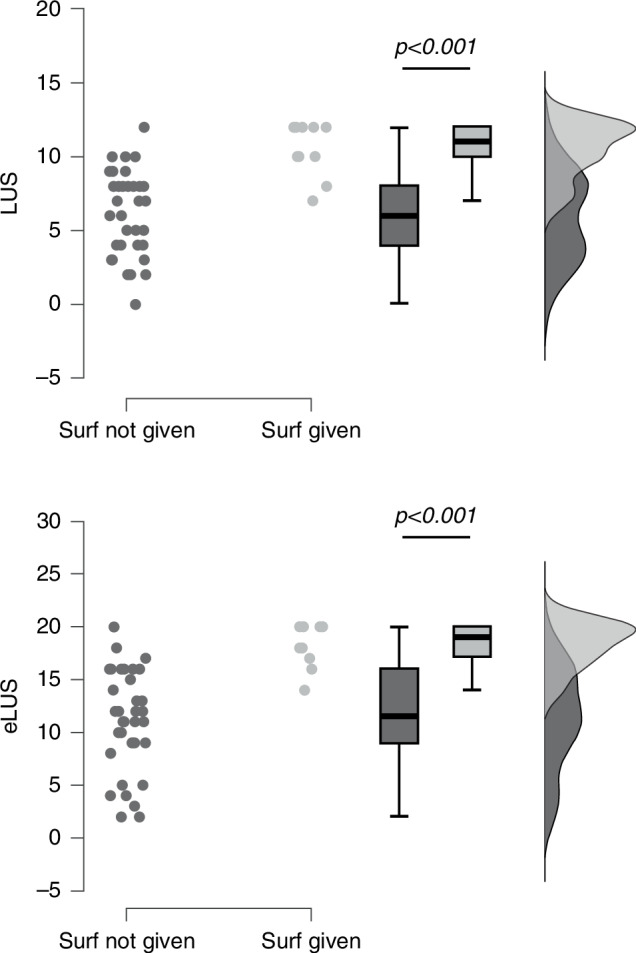
Ultrasound-assessed lung aeration. Aeration is depicted in terms of lung ultrasound score (LUS, top panel) and extended lung ultrasound score (eLUS, bottom panel). Each dot represents a neonate; box plots depict (from top to bottom) 95^th^, 75^th^, 50^th^, 25^th^, and 5^th^ percentiles. The raincloud curves on the right side represent the density (distributions) of datapoints using two different grey tones. Data were analyzed with Mann–Whitney test. Ultrasound scores are dimensionless variables. eLUS extended lung ultrasound score, LUS lung ultrasound score, Surf surfactant.

Ultrasound-assessed lung aeration was better in neonate who did not receive surfactant (Fig. 2). In fact, both lung ultrasound scores were consistently lower in neonate who were not treated with surfactant (LUS = 6 [4–8]; eLUS = 11 [9–16]) than in those who were (LUS = 10 [10–12], *p* < 0.001; eLUS = 19 [17–20], *p* < 0.001). OSI (LUS: ρ = 0.368, *p* = 0.01, adj-*r* = 0.460, *p* = 0.001; eLUS: ρ = 0.390, *p* = 0.006, adj-*r* = 0.427, *p* = 0.003) and SpO_2_/FiO_2_ ratio (LUS: ρ = -0.399, *p* = 0.005, adj-*r* = −0.503, *p* < 0.001; eLUS: ρ = −0.435, *p* = 0.002, adj-*r* = -0.483, *p* < 0.001) significantly correlated with both ultrasound scores. No other significant correlations were observed.

**Fig. 3:**
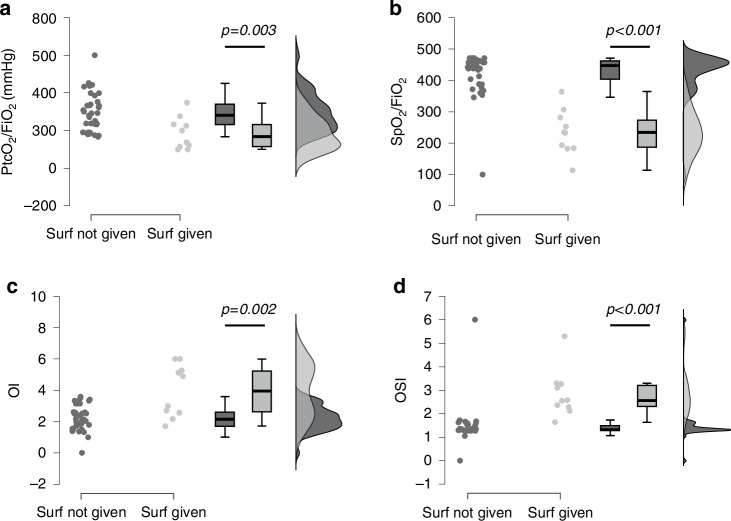
Oxygenation. Panels **a**–**d** depict results in terms of PtcO_2_/FiO_2_ ratio, SpO_2_/FiO_2_ ratio, oxygenation index (OI) and oxygen saturation index (OSI), respectively. Each dot represents a neonate; box plots depict (from top to bottom) 95^th^, 75^th^, 50^th^, 25^th^, and 5^th^ percentiles. The raincloud curves on the right side represent the density (distributions) of datapoints using two different grey tones. Data were analyzed with Mann–Whitney test. SpO_2_/FiO_2_ ratio, OI and OSI are dimensionless variables. FiO_2_ inspired oxygen fraction, OI oxygenation index, OSI oxygen saturation index, PtcO_2_ transcutaneous partial pressure of oxygen, SpO_2_ pre-ductal peripheral hemoglobin oxygen saturation, Surf surfactant.

PtcCO_2_ was similar between the two groups (45 [39–53] mmHg *versus* 54 [43–59] mmHg, *p* = 0.177). Oxygenation was better (Fig. 3) in neonates who eventually did not receive surfactant (PtcO_2_/FiO_2_ ratio: 281 [211–346]; SpO_2_/FiO_2_ ratio: 445 [388–462]; OI = 2.1 [1.6–2.6]; OSI = 1.3 [1.3–1.5]) than in those who did (PtcO_2_/FiO_2_ ratio: 168 [110–244], *p* = 0.003; SpO_2_/FiO_2_ ratio: 233 [183–288], *p* < 0.001; OI = 3.9 [2.5–5.4], *p* = 0.002; OSI = 2.6 [2.2–3.3], *p* < 0.001).

## Discussion

We offer novel insights about respiratory pathophysiology of late preterm and term neonates with respiratory failure in the first hours of life. Firstly, we observed that alveolar fluid may not be increased in neonates treated with surfactant; further, these patients may have significantly reduced lung aeration, and, finally, this reduction impairs oxygenation and does not seem to influence CO_2_ clearance.

The logical consequence of these findings would be that, in late preterm and term neonates receiving surfactant, the lung aeration is decreased not because of lung fluid, but rather for the reduced alveolar opening due to insufficient surfactant. This is consistent with recent literature hypothesizing that in term neonates, when alveolar volume is reduced, it would not be possible to accumulate excessive fluid in the alveoli.^4,34^ Other mechanisms, such as lung tissue inflammation or secondary surfactant dysfunction, do not play a role in our population. In fact, we excluded neonates recruited in the original study with respiratory failure whose lung fluid has an inflammatory origin and a different composition (e.g., aspirations, infections).^11^ This leaves the two most common neonatal respiratory failure mechanisms,^3,4^ namely surfactant insufficiency and excessive alveolar fluid accumulation, as the possible ones.

The lung aeration negatively correlates with oxygenation while it may have no effect on CO_2_. These findings are expected as oxygenation and lung aeration are logically worse in neonates needing surfactant and are consistent with previous data on extra-vascular lung water, lung aeration and gas exchange during the evolution of respiratory failure.^11,21,30,35–41^ Nonetheless, our findings add to the knowledge since this was the first study specifically investigating late preterm and term neonates based on surfactant treatment and using the three techniques to simultaneously assess extravascular lung water, lung aeration and gas exchange. Of note: Yoon et al.’s work was the only study with a similar design and reported LUS and TFC values similar to ours^36^; furthermore, quantitative lung ultrasound has been used in several projects, and the original analysis of our population demonstrated its accuracy to guide surfactant replacement.^11^ Finally, we used complex metrics to accurately describe oxygenation and this was not done before in late preterm and term neonates.

A formal comparison between LUS and oxygenation metrics would be biased since the criterion to decide surfactant administration (i.e. the FiO_2_ threshold) is included in the metrics calculation and therefore creates the so called “verification bias by incorporation”.^42^ However, in preterm neonates, the available data suggest that OI is less accurate than LUS to predict surfactant need.^33,43^ Anyway, the three used techniques give complementary information from different steps of the pathophysiology cascade, thus their joint evaluation provides a more comprehensive picture of respiratory failure in each neonate.

Investigating mixed populations of late preterm and term neonates with respiratory failure of any type (i.e. including more complex and infrequent lung injuries that were excluded here) would be interesting but need a different design, a larger sample size and was beyond our scope. Some preliminary data are however available from the literature. In fact, electrical cardiometry is able to detect the excessive fluid accumulation leading to TTN and also to predict the need for long-lasting ventilation in preterm infants unresponsive to surfactant replacement.^21,44^

Our limitations include the relatively small sample size, the use of point-of-care techniques, the clinical peculiarities of our population, the lack of surfactant assessment and the unique sampling which did not consider the evolution of these measurements overtime. The sample size may be prone to the presence of unnoticed confounders but is relatively similar to that of other studies in the field.^21,44^ One patient with clinically diagnosed TTN later deteriorated and received surfactant, so it is included in the surfactant-treated group: however, excluding that infant did not change the main findings. Point-of-care quantitative lung ultrasound and electrical cardiometry were chosen for their reliability, non-invasiveness and integration in our clinical routine. More accurate and invasive techniques (such as advanced hemodynamic monitoring, serial gas exchange evaluation based on arterial cannulation or lung mechanics assessments) might have deepened the pathophysiology assessment but are unfeasible or unethical during routine care of this population. We could not assess endogenous surfactant pool to complete the evaluation of the whole pathophysiology cascade: this is not always feasible in routine newborn care and may require invasive techniques which may also present ethical issues. Thus, our findings should be considered relatively accurate and add to our current knowledge. We focused on late preterm and term neonates who have been evaluated only once in early life: nonetheless, it is unlikely that repeated measurements would have provided different results since TFC and LUS usually decrease overtime,^21,35,38^ and surfactant was administered early within the first few hours of life.

## Conclusions

Late preterm and term neonates with respiratory failure (without lung tissue inflammation), treated with surfactant show decreased ultrasound-assessed lung aeration associated with impaired oxygenation likely due to surfactant insufficiency rather than excessive extra-vascular lung water.

## Data Availability

The datasets analyzed during the present work are available from the corresponding author on reasonable request, respecting all relevant regulations.
